# Prolyl hydroxylase domain enzyme PHD2 inhibits proliferation and metabolism in non-small cell lung cancer cells in HIF-dependent and HIF-independent manners

**DOI:** 10.3389/fonc.2024.1370393

**Published:** 2024-06-27

**Authors:** Hongyan Deng, Zixuan Wang, Chunchun Zhu, Zhu Chen

**Affiliations:** ^1^ Institute of Hydrobiology, Chinese Academy of Sciences, Wuhan, China; ^2^ College of Life Science, Wuhan University, Wuhan, China; ^3^ Department of Reproduction, Maternal and Child Health Hospital of Hubei Province, Tongji Medical College, Huazhong University of Science and Technology, Wuhan, China

**Keywords:** PHD2, prolyl hydroxylase, non-small cell lung cancer, HIF-dependent, HIF-independent

## Abstract

Prolyl hydroxylase domain protein 2 (PHD2) is one of the intracellular oxygen sensors that mediates proteasomal degradation of hypoxia-inducible factor (HIF)-α via hydroxylation under normoxic conditions. Because of its canonical function in the hypoxia signaling pathway, PHD2 is generally regarded as a tumor suppressor. However, the effects of PHD2 in tumorigenesis are not entirely dependent on HIF-α. Based on analysis of data from the Cancer Genome Atlas (TCGA) database, we observed that the expression of *PHD2* is upregulated in non-small cell lung cancer (NSCLC), which accounts for approximately 80–85% of lung cancers. This suggests that PHD2 may play an important role in NSCLC. However, the function of PHD2 in NSCLC remains largely unknown. In this study, we established *PHD2*-deficient H1299 cells and *PHD2*-knockdown A549 cells to investigate the function of PHD2 in NSCLC and found that PHD2 suppresses cell proliferation and metabolism but induces ROS levels in human NSCLC cells. Further results indicated that the function of PHD2 in NSCLC is dependent on its enzymatic activity and partially independent of HIF. Moreover, we performed RNA-sequencing and transcriptomic analysis to explore the underlying mechanisms and identified some potential targets and pathways regulated by PHD2, apart from the canonical HIF-mediated hypoxia signaling pathway. These results provide some clues to uncover novel roles of PHD2 in lung cancer progression.

## 1 Introduction

Prolyl hydroxylase domain protein 2 (*PHD2*, also known as egg-laying defective nine 1, *EGLN1*) belongs to the family of prolyl hydroxylase domain proteins (PHDs). The PHD family is an evolutionarily conserved superfamily of dioxygenases, consisting of four members: *PHD1* (*EGLN2*), *PHD2* (*EGLN1*), *PHD3* (*EGLN3*), and the less studied *PHD4* (*P4H-TM*, *EGLN4*) (1–5). The hydroxylation reaction catalyzed by PHDs is dependent on their conserved C-terminal catalytic domain, oxygen (O_2_), ferrous iron (Fe^2+^), 2-oxoglutarate (2-OG), and ascorbic acid (vitamin C) (6). PHDs act as intracellular oxygen sensors that mediate the proteasomal degradation of hypoxia-inducible factor (HIF)-α via hydroxylation under normoxic conditions (7, 8). HIF-α is expressed in almost all cell types and directly or indirectly regulates the expression of more than 100 genes (9). The HIF-mediated hypoxia signaling pathway is involved in various biological processes, such as metabolism, angiogenesis, tumorigenesis, and immunity (10–14). Increased expression of HIF-α has been reported in the majority of tumor cell types and has been correlated with poor patient prognosis (15). HIF-α promotes tumor progression by regulating the expression of key target genes (16), as HIF-1α supports tumor angiogenesis by enhancing the expression of vascular endothelial growth factor (*VEGF*) (17), and it also facilitates glucose uptake and glycolysis by upregulating the expression of glucose transporter 1 (*GLUT1*) and related genes (18).

Due to its canonical function in the hypoxia signaling pathway, PHD2 is generally considered to be a tumor suppressor (3, 6). However, the effects of PHD2 in tumorigenesis are not entirely dependent on HIF-α. The tumor-suppressing or tumor-promoting function of PHD2 may depend on its different targets in different tumors (19, 20). In gastric cancer and prostate cancer, PHD2 affects tumor development by inhibiting the HIF-mediated hypoxia signaling pathway (21–23). PHD2 suppresses neoplastic growth in colon cancer and breast cancer by attenuating NF-κB activity (24, 25). PHD2 inhibits breast cancer growth by hydroxylating B55α upon glucose starvation (26). PHD2 regulates EGFR stability and subsequent signaling in breast cancer cells (27). PHD2 acts in a hydroxylase-dependent manner to inhibit liver tumor cell proliferation and cyclin D1 expression (28). However, PHD2 promotes cancer progression via hydroxylation of Carabin in B-cell lymphoma (29). Lung cancer ranks among the most common and dangerous malignancies. Based on histologic type, lung cancer can be divided into non-small cell lung cancer (NSCLC) and small cell lung cancer (SCLC), with NSCLC accounting for 80–85% of cases (30, 31). Based on the data obtained from the Cancer Genome Atlas (TCGA) database, we found that *PHD2* expression is upregulated in lung cancer, suggesting that PHD2 may play a crucial role in lung cancer. However, the function of PHD2 in NSCLC remains largely unknown.

In this study, we generated *PHD2*-deficient H1299 cells and *PHD2*-knockdown A549 cells to elucidate the function of PHD2 in NSCLC, and found that PHD2 suppressed cell proliferation and metabolism, but induced ROS levels in human NSCLC cells. The function of PHD2 in NSCLC is dependent on its enzymatic activity and partially independent of HIF. To further explore the mechanism of PHD2 function in NSCLC, we performed RNA-seq and transcriptomic analysis, and identified some potential targets and pathways regulated by PHD2, apart from the canonical HIF-mediated hypoxia signaling pathway. These results provide some clues to discover novel roles of PHD2 in lung cancer progression.

## 2 Materials and methods

### 2.1 Cell line and culture condition

H1299, A549 and HEK293T cells, initially obtained from the American Type Culture Collection (ATCC), were cultivated in Dulbeccos’ modified Eagle medium (DMEM) (VivaCell Biosciences) supplemented with 10% fetal bovine serum (FBS) and maintained at 37°C in a humidified incubator containing 5% carbon dioxide (CO_2_).

### 2.2 Generation of H1299 or A549 knockout/knockdown cell lines

For the generation of H1299 or A549 knockout/knockdown cell lines targeting the indicated genes, the sgRNA sequence was inserted into the Lenti-CRISPRv2 vector and then transfected into HEK293T cells together with viral packaging plasmids (psPAX2 and pMD2G). After transfection, the medium was changed 6 hours later, and the viral supernatant was harvested and filtered through a 0.45 μm strainer. H1299 or A549 cells were then infected with viral supernatant and selected using 1 μg/ml puromycin for 2 weeks. The sequence of the sgRNA targeting *PHD2* is: CCCGCCGCTGTCATTGGCCA. The sequence of the sgRNA targeting *ARNT* is: GTCGCCGCTTAATAGCCCTC.

### 2.3 Generation of gene stably overexpressed cell lines

HEK293T cells were individually transfected with pHAGE empty, pHAGE-PHD2 or pHAGE-PHD2-H313A together with the packaging plasmids (psPAX2 and pMD2G). After 8 hours, the medium was removed and replaced with fresh medium containing 10% FBS, 10 μM β-mercaptoethanol, and 1% streptomycin-penicillin. Forty hours later, the supernatants were harvested, filtered through a 0.45 μm strainer, and subsequently applied to infect *PHD2*-deficient H1299 cells (*PHD2* ^-/-^) or *ARNT*-deficient H1299 cells (*ARNT* ^-/-^).

### 2.4 Western blot analysis and co-immunoprecipitation assay

Western blot analysis was performed as described previously (32). Antibodies comprising anti-PHD2 (#4835, Cell Signaling Technology), anti-HIF1α (#36169, Cell Signaling Technology), and anti-GAPDH antibody (#SC-47724, Santa Cruz Biotechnology) were used to detect the indicated proteins. The blots were photographed using a Fuji Film LAS4000 mini-luminescent image analyzer.

For the Co-immunoprecipitation assay, HEK293T cells were transfected with Myc-HIF-1α together with Flag-PHD2 or Flag empty vector as a control. Immunoprecipitation was performed using anti-Flag antibody-conjugated agarose beads (#A2220, Sigma), and the interaction was analyzed by Western blot analysis with anti-Myc (#sc-40, Santa Cruz) and anti-Flag (#F1804, Sigma) antibodies.

### 2.5 Quantitative real-time PCR assay

RNAiso Plus (TaKaRa Bio., Beijing, China) was used for total RNA extraction according to the manufacturer’s protocol. Revert Aid First Strand cDNA Synthesis Kit (Thermo Scientific, Waltham, MA, USA) was utilized for cDNA synthesis. MonAmpTM SYBR® Green qPCR Mix (high Rox) (Monad Bio., Shanghai, China) was employed for quantitative real-time PCR (qPCR) assays. The following primers were used: 5′-GATGCTAAAGCTATTTATGACT-3′ and 5′-GGAATGACATCATTGTGTCGG-3′ for *PDK1*; 5′- TGGCTTCTGGCATACCTGCT-3′ and 5′-GCTGCTTTCAGGACCACAGCT-3′ for *PGK1*; 5′-TGTGGGCCTTTTCGTTAACC-3′ and 5′-ATCATCAGCATTGAATTCCGC-3′ for *GLUT1*; 5′- AGGAAGAACAGACCCCCCAG-3′ and 5′-CAGCACCAACCCCAACAACT-3′ for *LDHA*; 5′- GAATTCCCAGTAAGTGGGGG-3′ and 5′-GGGCAGGGACTTAATCAACG-3′ for *18SrRNA*.

### 2.6 Cell proliferation and colony formation assay

For the cell proliferation assay, H1299 cells were seeded in 96-well plates at a density of 1 × 10^3^ cells per well and cultured for the indicated days. The CCK-8 assay was performed to assess cell growth rate following the manufacturer’s instructions.

For the colony formation assay, H1299 cells were seeded in 6-well plates at a density of 1 × 10^3^ cells per well and cultured for the indicated days. After seven days, colonies were fixed with methanol, stained with crystal violet (0.5% in methanol), rinsed with PBS, and then photographed. The colonies of appropriate size were individually counted from the images in each well.

### 2.7 Mitochondrial stress test and glycolytic rate test assays

The indicated H1299 cells (4 × 10^4^ cells/well) were cultivated in XF24 cell culture microplate (#102340–100, Agilent Technologies). The XF Cell Mito Stress Test kit (#103015–100, Agilent Technologies) was used in the mitochondrial stress test assay to assess the oxygen consumption rate (OCR) using the Seahorse XFe24 Extracellular Flux Analyzer (Agilent Technologies, Santa Clara, CA, USA). The XF Glycolytic Rate Assay Kit (#103344–100, Agilent Technologies) was used in the glycolytic rate test assay to assess the proton efflux rate (PER) using the Seahorse XFe24 Extracellular Flux Analyzer (Agilent Technologies, Santa Clara, CA, USA). Mitochondrial respiration, including basal respiration, maximal respiration, and spare respiratory capacity, were determined in the mitochondrial stress test assay by adding oligomycin (1.5 μM), carbonyl cyanide-4-(trifluoromethoxy) phenylhydrazone (FCCP, 2 μM), and antimycin A and rotenone mixture (0.5 μM) to the cell culture plate. Glycolytic flux, including basal glycolysis and compensatory glycolysis, were determined in the glycolytic rate test assay by adding antimycin A and rotenone mixture (0.5 μM) and 2-deoxy-D-glucose (50 mM) to the cell culture plate. Normalization to total cellular protein is used for the normalization of cells.

### 2.8 Glucose uptake assay

The fluorescent glucose analog 2-NBDG was used to evaluate glucose uptake. *PHD2*-deficient or wild-type H1299 cells (*PHD2* ^−/−^ or *PHD2* ^+/+^) were grown in glucose-free DMEM medium for approximately 6 hours. After the addition of 50 μM 2-NBDG, the cells were incubated in the dark at 37°C for 1 hour, and then the amount of 2-NBDG taken up by the cells was monitored by fluorescence microscopy or quantified by flow cytometry analysis (FACS).

### 2.9 Mitochondrial and intracellular ROS assay

MitoSOX™ Red (mitochondrial superoxide indicator) (#M36008, Thermo Fisher Scientific) and CM-H_2_DCFDA (general oxidative stress indicator) (#C6827, Thermo Fisher Scientific) were used to determine mitochondrial and intracellular ROS levels, respectively. After cell harvest, cells were incubated with 5 μM MitoSOX™ Red for 10 minutes at 37°C for the mitochondrial ROS assay, and with 1 μM CM-H_2_DCFDA for 1 hour at 37°C for the intracellular ROS assay.

### 2.10 RNA-Seq and bioinformatics analysis

Total RNA from *PHD2*-deficient or wild-type H1299 cells (*PHD2* ^−/−^ or *PHD2* ^+/+^) was purified using the RNeasy Mini Kit (QIAGEN, 74104). Sequencing libraries were prepared using the NEBNext Ultra RNA Library Prep Kit for Illumina (NEB, USA, #E7530L) according to the manufacturer’s protocol. Qualified libraries were pooled and sequenced on Illumina NovaSeq platforms using the PE150 strategy at Wuhan Benagen Technology Co., Ltd. (Wuhan, China). Fastp version 0.21.0 (https://github.com/OpenGene/fastp) was used to perform basic statistics on the quality of the raw reads. The clean reads were mapped to the human reference genome (hg38) using Star version 2.7.9a (https://github.com/alexdobin/STAR). RSEM version 1.3.3 (https://github.com/deweylab/RSEM) was used to calculate gene and transcript expression levels expressed as fragments per kilobase of transcript per million fragments mapped (FPKM).

Gene expression levels in samples from *PHD2*-deficient H1299 cells were compared to those in samples from wild-type H1299 cells to identify differentially expressed genes (DEGs). DEGs were identified based on the following parameters: log_2_ (fold change) ≥1 and *P* value <0.05. The heatmap of the expression changes of the indicated genes and the volcano plot of the DEGs were generated using ImageGP (https://www.bic.ac.cn/BIC/). Gene Ontology (GO) enrichment analysis for the DEGs was performed using the clusterProfiler version 3.14.3 (http://www.bioconductor.org/packages/release/bioc/html/clusterProfiler.html). Kyoto Encyclopedia of Genes and Genomes (KEGG) enrichment analysis was performed using Gene Set Enrichment Analysis (GSEA) software (http://software.broadinstitute.org/gsea/index.jsp). The original RNA-seq data were uploaded to the GEO DataSets.

### 2.11 Immunofluorescence confocal microscopy

Cells were grown on glass coverslips overnight, and then fixed with 4% paraformaldehyde in PBS for 20 min, permeabilized with 0.1% Triton X‐100, and blocked with 1% bovine serum albumin. Then, the cells were stained with anti-Ki67 primary antibody (#Ab16667, Abcam) followed by incubation with fluorescent‐dye‐conjugated secondary antibody. Nuclei were counterstained with DAPI (Sigma‐Aldrich). Imaging of the cells was carried out using a Leica laser‐scanning confocal microscope.

### 2.12 Statistical analysis

All statistical analyses were performed using GraphPad Prism software (8.3.0). Results with error bars represent mean ± SD. Statistical analysis was based on Student’s two-tailed *t*-test. A *p*-value less than 0.05 was regarded as significant. Statistical significance is presented as follows: * *p*< 0.05, ** *p*<0.01, *** *p*<0.001, **** *p*<0.0001.

## 3 Results

### 3.1 *PHD2* deficiency enhances proliferation and metabolism in H1299 cells

**Figure 1:**
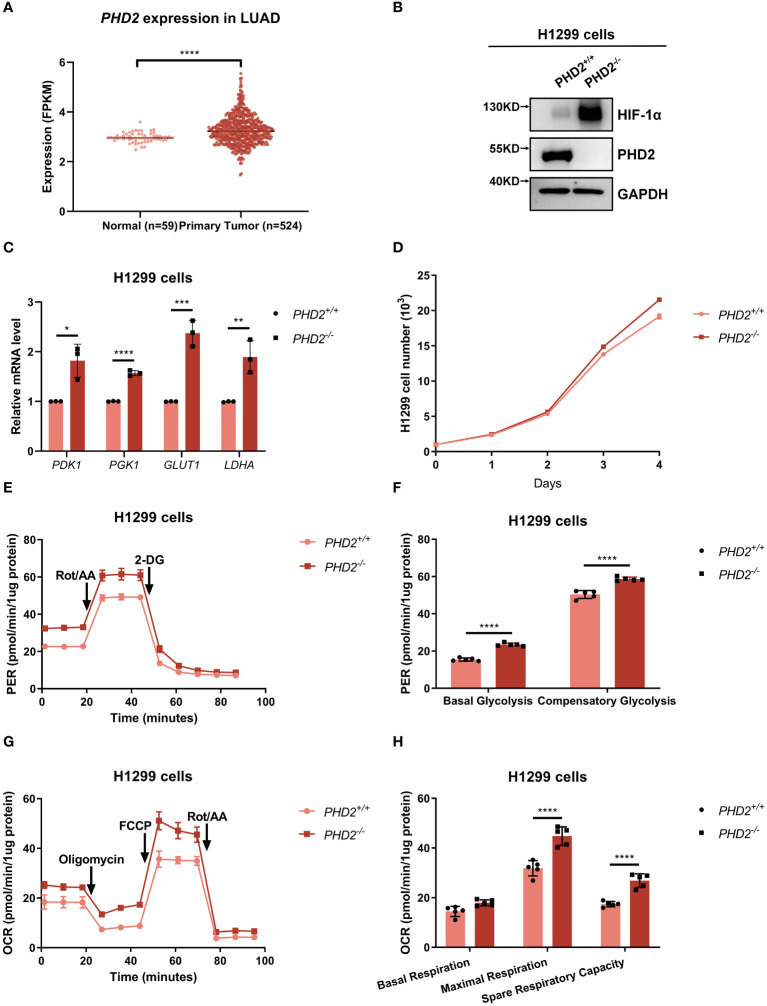
*PHD2* deficiency enhances proliferation and metabolism in H1299 cells. **(A)** Expression data of *PHD2* in lung cancer tissues (n = 515) and adjacent normal tissues (n = 59) were obtained from The Cancer Genome Atlas (TCGA) data (https://cancergenome.nih.gov/). The mRNA levels of *PHD2* were higher in tumors than in normal tissues, as determined by Student’s *t*-test; ****p<0.0001. **(B)** Western blot analysis of the indicated protein levels in *PHD2*-deficient or wild-type H1299 cells (*PHD2^−/−^* or *PHD2^+/+^*). **(C)** Quantitative real time PCR (qPCR) analysis of *PDK1*, *PGK1*, *GLUT1*, and *LDHA* mRNA in *PHD2*-deficient or wild-type H1299 cells (*PHD2^−/−^* or *PHD2^+/+^*). Data show mean ± SD; Student’s two-tailed *t*-test; *p<0.05, **p<0.01, ***p<0.001, ****p < 0.0001; data from 3 independent experiments. **(D)** Growth curves of *PHD2*-deficient or wild-type H1299 cells (*PHD2^−/−^* or *PHD2^+/+^*) (n = 3) cultivated for the indicated days and analyzed by CCK-8 assay. **(E, F)** Changes in proton efflux rate (PER) in *PHD2*-deficient or wild-type H1299 cells (*PHD2^−/−^* or *PHD2^+/+^*) (n = 5) determined using the Seahorse XFe24 Extracellular Flux Analyzer. Statistics of compensatory glycolysis and basal glycolysis are shown in **(F)**. **(G, H)** Changes in oxygen consumption rate (OCR) in *PHD2*-deficient or wild-type H1299 cells (*PHD2^−/−^* or *PHD2^+/+^*) (n = 5) determined using the Seahorse XFe24 Extracellular Flux Analyzer. Statistics of the capacity of maximal respiration, basal respiration, and spare respiratory are shown in **(H)**.

Based on lung adenocarcinoma (LUAD) data extracted from the Cancer Genome Atlas (TCGA) database, we found that *PHD2* expression was higher in lung cancer tissues than in adjacent normal tissues (**Figure 1A**), indicating an essential role of PHD2 in non-small cell lung cancer (NSCLC). Therefore, we knocked out *PHD2* in human NSCLC H1299 cells using CRISPR/Cas9 technology. Since PHD2 interacts with HIF-1α (**Supplementary Figure S1A**), and PHD2-mediated hydroxylation leads to the degradation of HIF-1α (**Supplementary Figures S1B, C**), the results of PHD2 and HIF-1α protein levels in **Figure 1B** indicated that *PHD2*-deficient H1299 cells were well established. In addition, increased mRNA levels of *PDK1*, *PGK1*, *GLUT1*, and *LDHA* indicated that PHD2 was efficiently disrupted in *PHD2*-deficient H1299 cells (**Figure 1C**).

**Figure 2:**
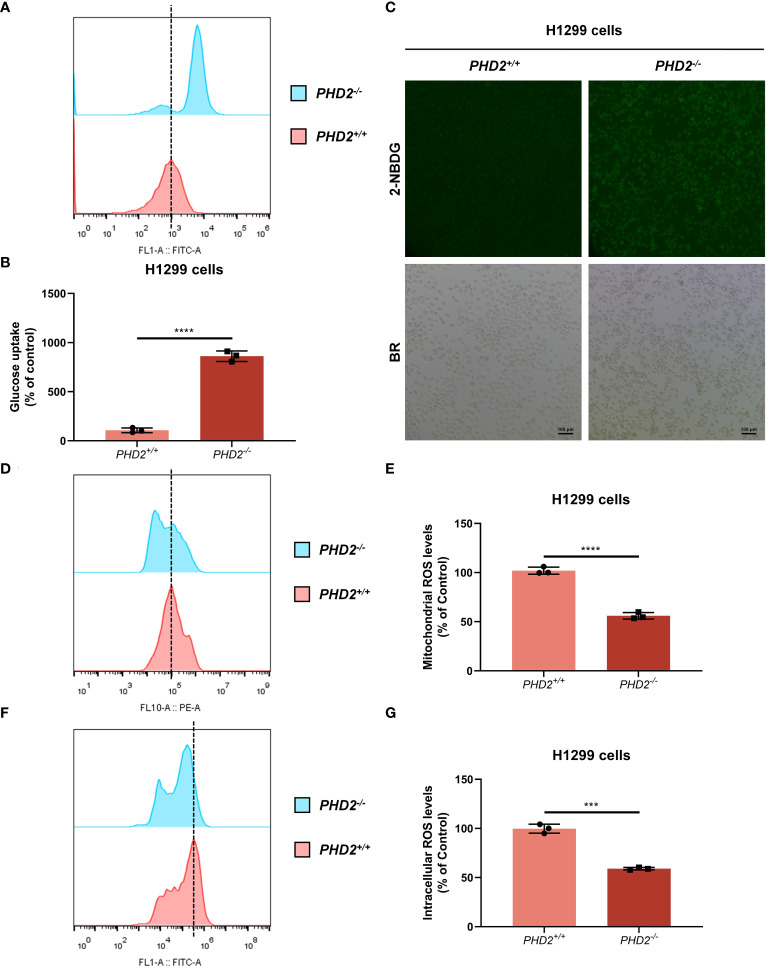
*PHD2* deficiency promotes glucose uptake and reduces reactive oxygen species (ROS) levels in H1299 cells. **(A–C)** Glucose uptake in *PHD2*-deficient or wild-type H1299 cells (*PHD2^−/−^* or *PHD2^+/+^*) (n = 3) was analyzed using the fluorescent glucose analog 2-NBDG and evaluated by flow cytometry analysis **(A, B)** and fluorescence microscopy **(C)**. **(D, E)** Mitochondrial ROS levels in *PHD2*-deficient or wild-type H1299 cells (*PHD2^−/−^* or *PHD2^+/+^*) were examined by flow cytometry analysis. Data represent mean ± SD; Student’s two-tailed *t*-test; ****p < 0.0001; data from 3 independent experiments. **(F, G)** Intracellular ROS levels in *PHD2*-deficient or wild-type H1299 cells (*PHD2^−/−^* or *PHD2^+/+^*) were examined by flow cytometry analysis. Data represent mean ± SD; Student’s two-tailed *t*-test; ***p < 0.001; data from 3 independent experiments.

We then examined the effect of *PHD2* deficiency in H1299 cells. Proliferation of *PHD2*-deficient H1299 cells was faster than that of wild-type H1299 cells (**Figure 1D**), and the results were confirmed by immunofluorescence staining with anti-Ki67 antibody in H1299 cells (**Supplementary Figure S2A**). Similar results were obtained in human NSCLC A549 cells (**Supplementary Figures S2B, C**). Furthermore, *PHD2* deficiency resulted in increased basal glycolysis and compensatory glycolysis based on proton efflux rate (PER) measurement (**Figures 1E, F**), while spare respiratory capacity and maximal respiration of *PHD2*-deficient H1299 cells were significantly higher than that of wild-type H1299 cells based on mitochondrial stress test (**Figures 1G, H**). Furthermore, glucose uptake was strongly induced in *PHD2*-deficient H1299 cells (**Figures 2A–C**). In addition, we evaluated the effect of PHD2 on the accumulation of reactive oxygen species (ROS) and found that mitochondrial ROS levels and intracellular ROS levels were significantly reduced in *PHD2*-deficient H1299 cells (**Figures 2D–G**). Taken together, these data suggest that PHD2 deficiency promotes proliferation and metabolism in NSCLC cells.

### 3.2 *PHD2* function in NSCLC cells depends on its enzymatic activity

**Figure 3:**
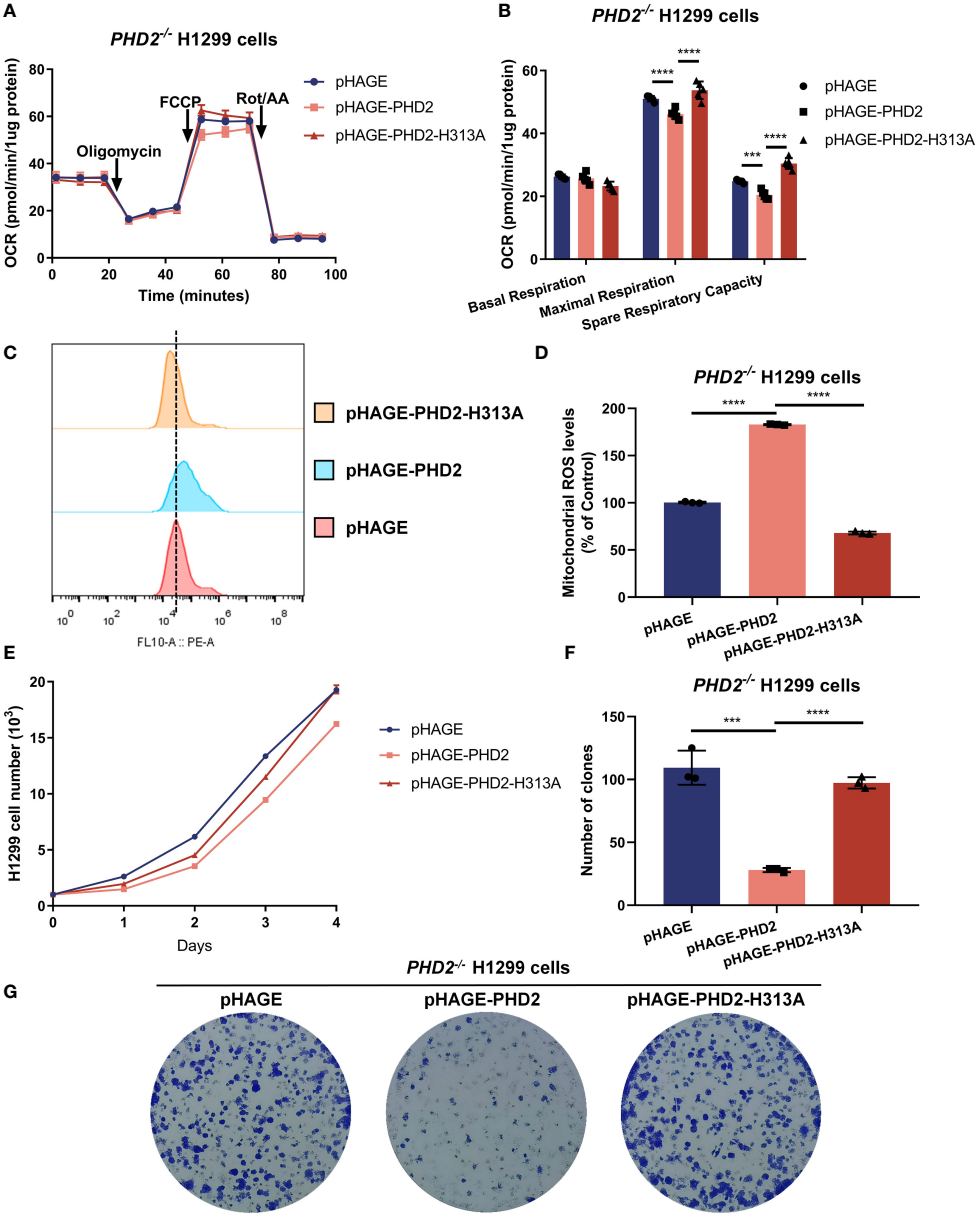
PHD2 impairs metabolism and proliferation in H1299 cells in a hydroxylase-dependent manner. **(A, B)** Seahorse XFe24 Extracellular Flux Analyzer was employed to assess the oxygen consumption rate (OCR) in *PHD2*-deficient H1299 cells (*PHD2^−/−^*) reconstituted with PHD2 or its enzymatically deficient mutant (H313A) by lentivirus (n = 5). Statistics of maximal respiration, basal respiration, and spare respiratory capacity are shown in **(B)**. **(C, D)** Mitochondrial ROS levels detected by flow cytometry analysis in *PHD2*-deficient H1299 cells (*PHD2^−/−^*) reconstituted with PHD2 or its enzymatically deficient mutant (H313A) by lentivirus. Data represent mean ± SD; Student’s two-tailed t-test; ***p < 0.001, ****p < 0.0001 data from 3 independent experiments. **(E)** Growth curves of *PHD2*-deficient H1299 cells (*PHD2^−/−^*) reconstituted with PHD2 or its enzymatically deficient mutant (H313A) by lentivirus (n = 3), cultured for the indicated days and analyzed by CCK-8 assay. **(F, G)** Colony formation of *PHD2*-deficient H1299 cells (*PHD2^−/−^*) reconstituted with PHD2 or its enzymatically deficient mutant (H313A) by lentivirus (n = 3), cultivated for 7 days, by colony formation assay. Data represent mean ± SD; Student's two-tailed t-test; ***p < 0.001, ****p < 0.0001; data from 3 independent experiments.

To assess whether the function of *PHD2* in NSCLC cells is dependent on its enzymatic activity, we reconstituted PHD2 or its enzymatically deficient mutant (H313A) in *PHD2*-deficient H1299 cells by lentivirus. As expected, the spare respiratory capacity and maximal respiration of *PHD2*-deficient H1299 cells reconstituted with PHD2 were lower than those of *PHD2*-deficient H1299 cells reconstituted with pHAGE empty vector, and the spare respiratory capacity and maximal respiration of *PHD2*-deficient H1299 cells reconstituted with PHD2 were lower than those of *PHD2*-deficient H1299 cells reconstituted with PHD2-H313A mutant (**Figures 3A, B**). Similarly, mitochondrial ROS levels in *PHD2*-deficient H1299 cells reconstituted with PHD2 were higher than those in *PHD2*-deficient H1299 cells reconstituted with pHAGE empty vector or PHD2-H313A mutant (**Figures 3C, D**). Similar results were obtained in the growth curve assay and the colony formation assay (**Figures 3E–G**). Taken together, these results suggest that PHD2 function in NSCLC cells is dependent on its enzymatic activity.

### 3.3 Function of PHD2 in NSCLC cells is at least partially independent of its canonical function in the hypoxia signaling pathway

**Figure 4:**
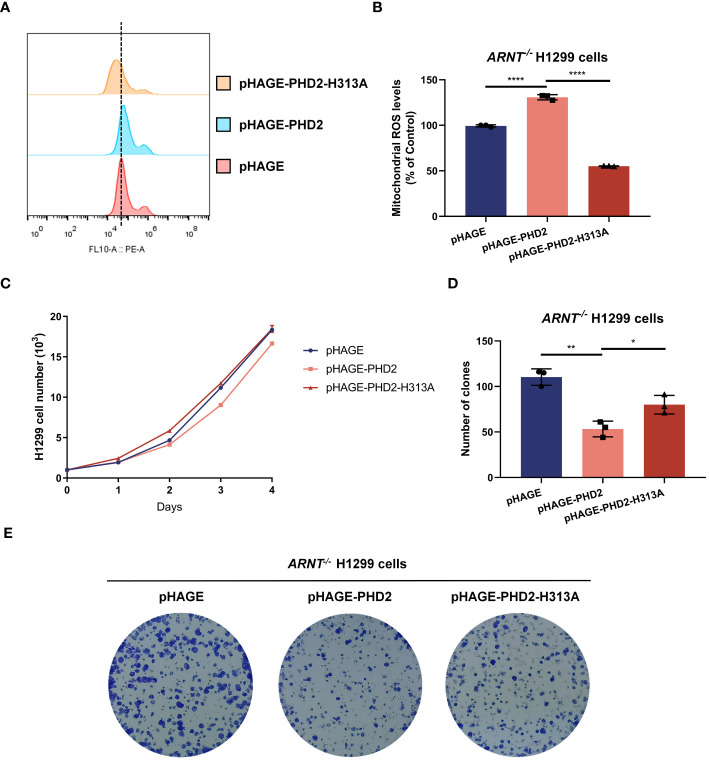
PHD2 reduces reactive oxygen species (ROS) levels and inhibits cell proliferation in H1299 cells in a hydroxylase-dependent but HIF-independent manner. **(A, B)** Mitochondrial ROS levels detected by flow cytometry analysis in *ARNT*-deficient H1299 cells (*ARNT^−/−^*) stably overexpressing PHD2 or its enzymatically deficient mutant (H313A) by lentivirus. Data show mean ± SD; Student’s two-tailed *t*-test; ****p < 0.0001; data from 3 independent experiments. **(C)** Growth curves of *ARNT*-deficient H1299 cells (*ARNT^−/−^*) with stably overexpressed PHD2 or its enzymatically deficient mutant (H313A) by lentivirus (n = 3) cultivated for the indicated days and analyzed by CCK-8 assay. **(D, E)** Colony formation of *ARNT*-deficient H1299 cells (*ARNT^−/−^*) with stably overexpressed PHD2 or its enzymatically deficient mutant (H313A) by lentivirus (n = 3) cultivated for 7 days by colony formation assay. Data represent mean ± SD; Student's two-tailed t-test; *p < 0.05, **p < 0.01; data from 3 independent experiments.

PHD2-mediated hydroxylation and subsequent ubiquitination and degradation of HIF-1α was considered to be the canonical and most important function of PHD2, and the above results indicated that *PHD2* deficiency indeed led to the stabilization of HIF-1α and the induction of HIF target genes. Therefore, we investigated whether the function of PHD2 in NSCLC cells is dependent on its canonical function in the hypoxia signaling pathway. We generated *ARNT*-deficient H1299 cells and established *ARNT*-deficient H1299 cells reconstituted with PHD2 or PHD2-H313A mutant by lentivirus. Stable overexpression with wild-type PHD2 resulted in higher mitochondrial ROS levels than that with pHAGE empty vector or PHD2-H313A mutant (**Figures 4A, B**). In addition, the proliferation rate of *ARNT*-deficient H1299 cells stably overexpressed with empty vector or PHD2-H313A was higher than that of *ARNT*-deficient H1299 cells overexpressed with PHD2, as shown by growth curve assay and colony formation assay (**Figures 4C–E**). The trend in these *ARNT*-deficient H1299 cells was consistent with that in the *ARNT*-sufficient H1299 cells above. Therefore, these data suggest that PHD2 function in NSCLC cells is at least partially independent of its canonical function in the hypoxia signaling pathway.

### 3.4 RNA-seq and transcriptomic analysis in *PHD2*-deficient or wild-type H1299 cells

**Figure 5:**
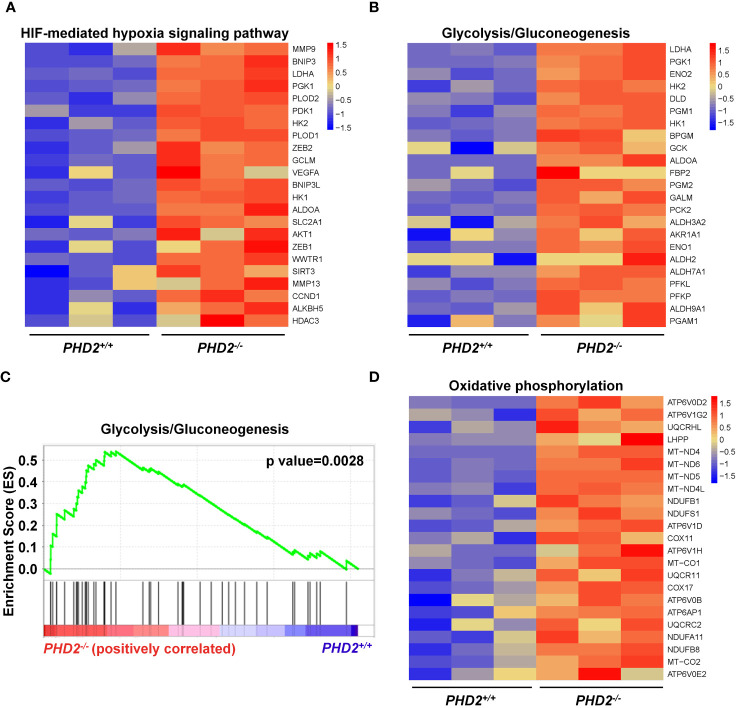
Enrichment of HIF target genes and metabolism-related genes in transcriptomic analysis in *PHD2*-deficient or wild-type H1299 cells. **(A)** Heatmap for expression of HIF target genes in *PHD2*-deficient or wild-type H1299 cells (*PHD2* ^−/−^ or *PHD2* ^+/+^). **(B)** Heatmap for expression of genes related to the glycolysis/gluconeogenesis pathway in *PHD2*-deficient or wild-type H1299 cells (*PHD2* ^−/−^ or *PHD2* ^+/+^). **(C)** GSEA enrichment plots of the glycolysis/gluconeogenesis pathway in *PHD2*-deficient or wild-type H1299 cells (*PHD2* ^−/−^ or *PHD2* ^+/+^). Red represents high expression. Blue represents low expression. **(D)** Heatmap for expression of genes related to the oxidative phosphorylation pathway in *PHD2*-deficient or wild-type H1299 cells (*PHD2* ^−/−^ or *PHD2* ^+/+^).

Since the function of PHD2 in NSCLC cells is partially independent of its canonical function in the hypoxia signaling pathway, we performed RNA-seq and transcriptomic analysis to explore the possible mechanisms of PHD2 functions in lung cancer. The heatmap showed that the expression of HIF target genes, such as *MMP9*, *BNIP3*, *LDHA*, etc., was upregulated in *PHD2*-deficient H1299 cells (**Figure 5A**). Moreover, the heatmap showed that the glycolysis/gluconeogenesis pathway-related genes were activated in *PHD2*-deficient H1299 cells (**Figure 5B**), and GSEA enrichment plots indicated that the glycolysis/gluconeogenesis pathway was significantly enriched in *PHD2*-deficient H1299 cells (**Figure 5C**). Some glycolysis/gluconeogenesis pathway-related genes overlapped with HIF target genes, such as *LDHA*, *PGK1*, *HK2*, etc., which may contribute to the fact that PHD2 can regulate the glycolysis pathway through the HIF-mediated signaling pathway. In addition, the heatmap showed that the genes related to the oxidative phosphorylation pathway were also induced in *PHD2*-deficient H1299 cells (**Figure 5D**).

**Figure 6:**
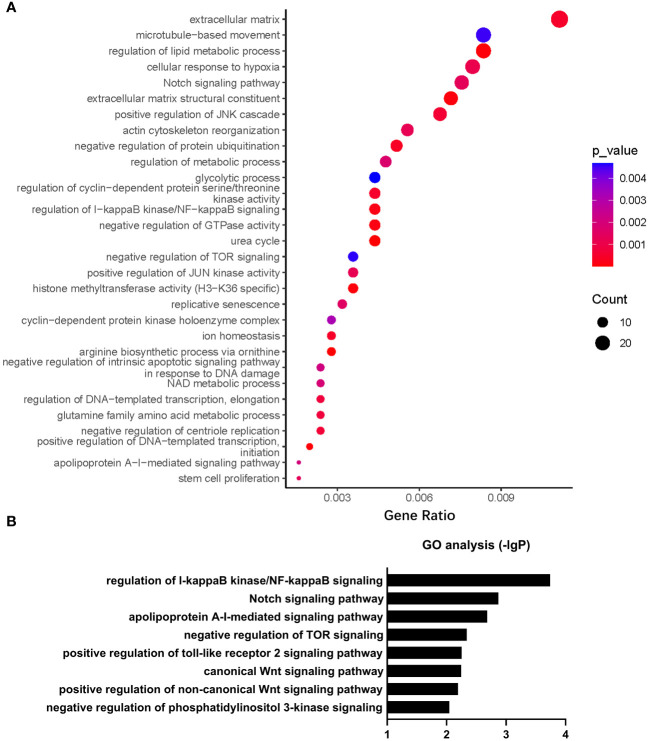
Gene Ontology (GO) enrichment of transcriptomic analysis in *PHD2*-deficient or wild-type H1299 cells. **(A, B)** Pathway enrichment of GO analysis in *PHD2*-deficient or wild-type H1299 cells (*PHD2* ^−/−^ or *PHD2* ^+/+^).

**Figure 7:**
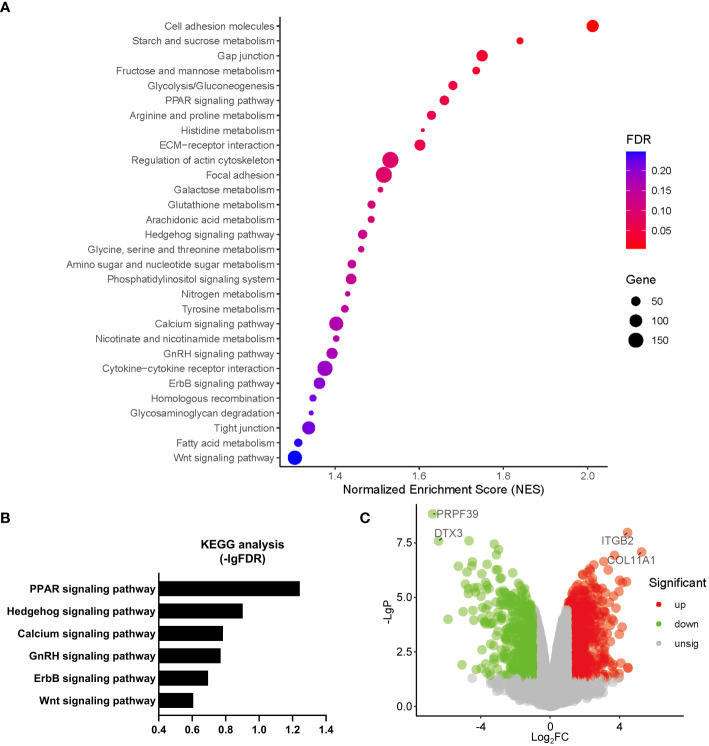
Kyoto Encyclopedia of Genes and Genomes (KEGG) enrichment of transcriptomic analysis in *PHD2*-deficient or wild-type H1299 cells. **(A, B)** Pathway enrichment of KEGG analysis in *PHD2*-deficient or wild-type H1299 cells (*PHD2* ^−/−^ or *PHD2* ^+/+^). **(C)** Volcano plot of differentially expressed genes (DEGs) in *PHD2*-deficient or wild-type H1299 cells (*PHD2* ^−/−^ or *PHD2* ^+/+^).

Gene Ontology (GO) analysis revealed that a number of biological processes, including extracellular matrix, cytoskeleton, metabolism, signal transduction, cell cycle, ion homeostasis, transcription, stem cell proliferation, etc., were enriched in *PHD2*-deficient H1299 cells (**Figure 6A**). Among these processes, the cellular response to hypoxia pathway was indeed enriched, confirming the activation of the hypoxia signaling pathway in *PHD2*-deficient H1299 cells (**Figure 6A**). Furthermore, we found that some important signaling pathways related to tumor progression, including NF-kappaB (NF-κB) signaling pathway, Notch signaling pathway, Wnt signaling pathway, and phosphatidylinositol 3-kinase (PI3K) signaling pathway, were enriched in *PHD2*-deficient H1299 cells (**Figure 6B**). Kyoto Encyclopedia of Genes and Genomes (KEGG) analysis revealed that several biological processes, including carbohydrate metabolism, amino acid metabolism, lipid metabolism, nitrogen metabolism, cell adhesion, gap junction, signal transduction, etc., were enriched in *PHD2*-deficient H1299 cells (**Figure 7A**). Similarly, we found that some important signaling pathways related to tumor progression, including PPAR signaling pathway, Hedgehog (Hh) signaling pathway, Calcium (Ca^2+^) signaling pathway, GnRH signaling pathway, ErbB signaling pathway, and Wnt signaling pathway, were enriched in *PHD2*-deficient H1299 cell (**Figure 7B**). The volcano plot showed that some genes, such as *ITGB2* and *COL11A1*, were highly upregulated in *PHD2*-deficient H1299 cells, while other genes, such as *PRPF39* and *DTX3*, were highly downregulated in *PHD2*-deficient H1299 cells (**Figure 7C**). These results demonstrated that PHD2 likely has multiple targets in lung cancer, providing clues for understanding the potential mechanisms of PHD2 function in lung cancer progression.

## 4 Discussion

In this study, we analyzed the data obtained from the Cancer Genome Atlas (TCGA) database and observed that *PHD2* expression was higher in lung cancer tissues than in adjacent normal tissues. Similar findings have been reported by other groups. Chen et al. investigated the mRNA levels of *PHD2* in 62 pairs of normal and NSCLC samples by RT-qPCR and found that *PHD2* expression was significantly higher in lung cancer tissues than in the adjacent normal tissues. Furthermore, *PHD2* expression was upregulated in 48 NSCLC patient samples (77.4% of the 62 samples) (33). However, Koren et al. investigated the expression of *PHD2* mRNA in 73 NSCLC primary tumors and 10 normal lung samples by RT-qPCR. The results showed that the expression of *PHD2* was significantly lower in lung cancer tissues than in normal tissues. Low expression of *PHD2* was associated with larger tumor size and worse prognosis in NSCLC patients (34). At present, there is no conclusion about the upregulation or downregulation of *PHD2* in lung cancer tissues. These different findings may be related to the variation in sample size or the discrepancy in tumor stage between different samples.

The enzymatic activity of PHD2 as a prolyl hydroxylase may deserve more attention than its expression level. Tumor cells generally proliferate rapidly and readily absorb oxygen and nutrients from blood vessels (35). However, as the tumor grows, the blood vessels become insufficient to supply the cells inside the tumor. The cells inside the tumor are often in a hypoxic state. The lack of oxygen results in the inhibition of the enzymatic activity of PHD2, leading to the activation of the HIF-mediated hypoxia signaling pathway, which promotes tumor proliferation, migration and angiogenesis. Alteration of the enzymatic activity of PHD2 is an essential event during tumor progression, while the expression of PHD2 is likely to change subsequently with tumor development. In this study, we demonstrated that the suppressive effect of PHD2 on NSCLC is entirely dependent on its enzymatic activity, as enzymatic deficiency of PHD2 completely abolished the inhibition of PHD2 on tumor proliferation and metabolism, as well as the induction of ROS levels. Therefore, modulation of PHD2 enzymatic activity with specific inhibitors may play vital roles in the treatment of NSCLC.

To explore the underlying mechanisms of PHD2 function in lung cancer progression, we performed RNA-seq and transcriptomic analysis in *PHD2*-deficient and wild-type H1299 cells. GO analysis showed that some important signaling pathways related to tumor progression, including NF-κB signaling pathway, Notch signaling pathway, TOR signaling pathway, Wnt signaling pathway, and PI3K signaling pathway, were enriched in *PHD2*-deficient H1299 cells. Meylan et al. showed that inhibition of the NF-κB signaling pathway can significantly reduce tumor growth in lung cancer (36). PHD1 and PHD3 have been reported to negatively regulate the NF-κB pathway through IκB kinase β (IKKβ) and heat shock protein 90 (Hsp90) (37–39). Takeda et al. showed that PHD2 negatively regulates NF-κB activity in macrophages (40). In addition, both Notch1 and Notch3 have been reported to enhance cell proliferation and suppress apoptosis in NSCLC cells (41, 42). The LKB1/AMPK/mTOR signaling pathway has been identified as a tumor suppressor axis in NSCLC (43–45). Notably, inhibition of PHD has been reported to result in the inability of glutaminolysis to activate mTORC1 (46–48). These results suggest that PHD plays an aggressive role in the activation of mTORC1. Moreover, in this study, KEGG analysis revealed that some important signaling pathways related to tumor progression, including PPAR signaling pathway, Hh signaling pathway, Ca^2+^ signaling pathway, GnRH signaling pathway, ErbB signaling pathway, and Wnt signaling pathway, were enriched in *PHD2*-deficient H1299 cell. Similarly, abnormal activation of canonical and non-canonical Wnt signaling pathway has been observed in NSCLC in previous studies. Wen YA et al. reported that silencing of *PHD2* rescued the expression of Wnt/β-catenin pathway target genes in colon cancer (49). Similarly, the activation of the Hh signaling pathway was found to be enhanced in lung cancer. The Hh signaling pathway appears to play a critical role in promoting cell proliferation and angiogenesis (50, 51), while PHD2 inhibition can activate the Hh signaling pathway via the GPT2-α-KG-PHD2 axis in breast cancer (52). The volcano plot showed the most up- and down-regulated genes in *PHD2*-deficient H1299 cells, which may play an important role in lung cancer progression. These results suggest that PHD2 may have multiple targets and function through multiple pathways to fulfill its role in lung cancer progression. Further identification of the key targets of PHD2 in lung cancer progression will shed light on the treatment of NSCLC.

In this study, we have provided evidence that PHD2 suppresses cell proliferation and metabolism, but induces ROS levels in human NSCLC cells. Compared with the heterogeneity of tumor tissues, the homogeneity of NSCLC cell lines makes our results relatively stable and reliable. However, the actual function of PHD2 in NSCLC requires further verification using mouse models or a large number of patient samples. The function of PHD2 in NSCLC is dependent on its enzymatic activity and partially independent of HIF. Transcriptomic analysis revealed some potential targets and pathways regulated by PHD2, providing some clues to discover some novel roles of PHD2 in lung cancer progression.

## Data availability statement

The datasets presented in this study can be found in online repositories. The names of the repository/repositories and accession number(s) can be found below: https://www.ncbi.nlm.nih.gov/geo/, GSE239389.

## Ethics statement

Ethical approval was not required for the studies on humans in accordance with the local legislation and institutional requirements because only commercially available established cell lines were used.

## Author contributions

HD: Conceptualization, Data curation, Investigation, Methodology, Visualization, Writing – original draft. ZW: Investigation, Visualization, Writing – original draft. CZ: Investigation, Visualization, Writing – original draft. ZC: Conceptualization, Investigation, Methodology, Supervision, Writing – original draft, Writing – review & editing.
