# Supplementary material for: Prolyl hydroxylase domain enzyme PHD2 inhibits proliferation and metabolism in non-small cell lung cancer cells in HIF-dependent and HIF-independent manners
Source: Front Oncol. 2024 Jun 27;14:1370393. doi: 10.3389/fonc.2024.1370393 (PMC11240288; doi:10.3389/fonc.2024.1370393)
Supplement: Supplementary file 1 [file DataSheet_1.docx]

**Supplementary information**

**Supplementary Data**

**
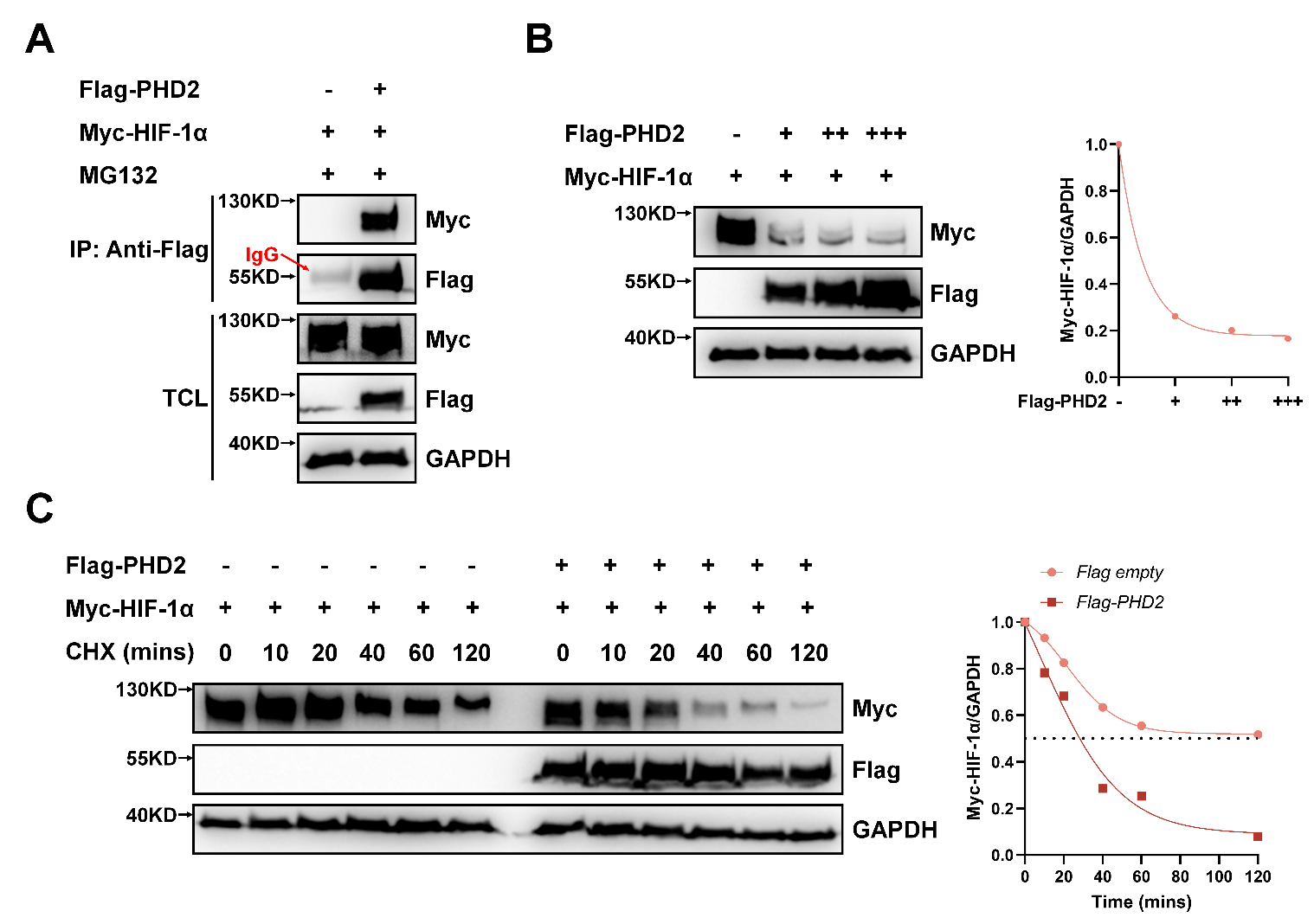
**

**Fig. S1. The interaction between PHD2 and HIF-1α and the degradation of HIF-1α by PHD2.**

(A) Co-immunoprecipitation analysis of Flag-PHD2 with Myc-HIF-1α. HEK293T cells were transfected with Myc-HIF-1α, together with Flag empty vector, or Flag-PHD2 for 24 h, followed by treatment with MG132 (20 μM) for 8 h. Anti-Flag-conjugated agarose beads were used for co-immunoprecipitation, and the indicated antibodies were used for detection.

(B) Western blot analysis of exogenous HIF-1α protein in HEK293T cells transfected with Myc-HIF-1α together with an increased amount of Flag-PHD2 for 24 h. The relative intensities of Myc-HIF-1α were determined by normalizing the intensities of Myc-HIF-1α to the intensities of GAPDH.

(C) Western blot analysis of exogenous HIF-1α protein in HEK293T cells transfected with Myc-HIF-1α together with Flag-PHD2 or Flag empty vector (control) for 24 h, followed by treatment with cycloheximide (CHX) (50 μg/ml) for the indicated time. The relative intensities of Myc-HIF-1α were determined by normalizing the intensities of Myc-HIF-1α to the intensities of GAPDH.


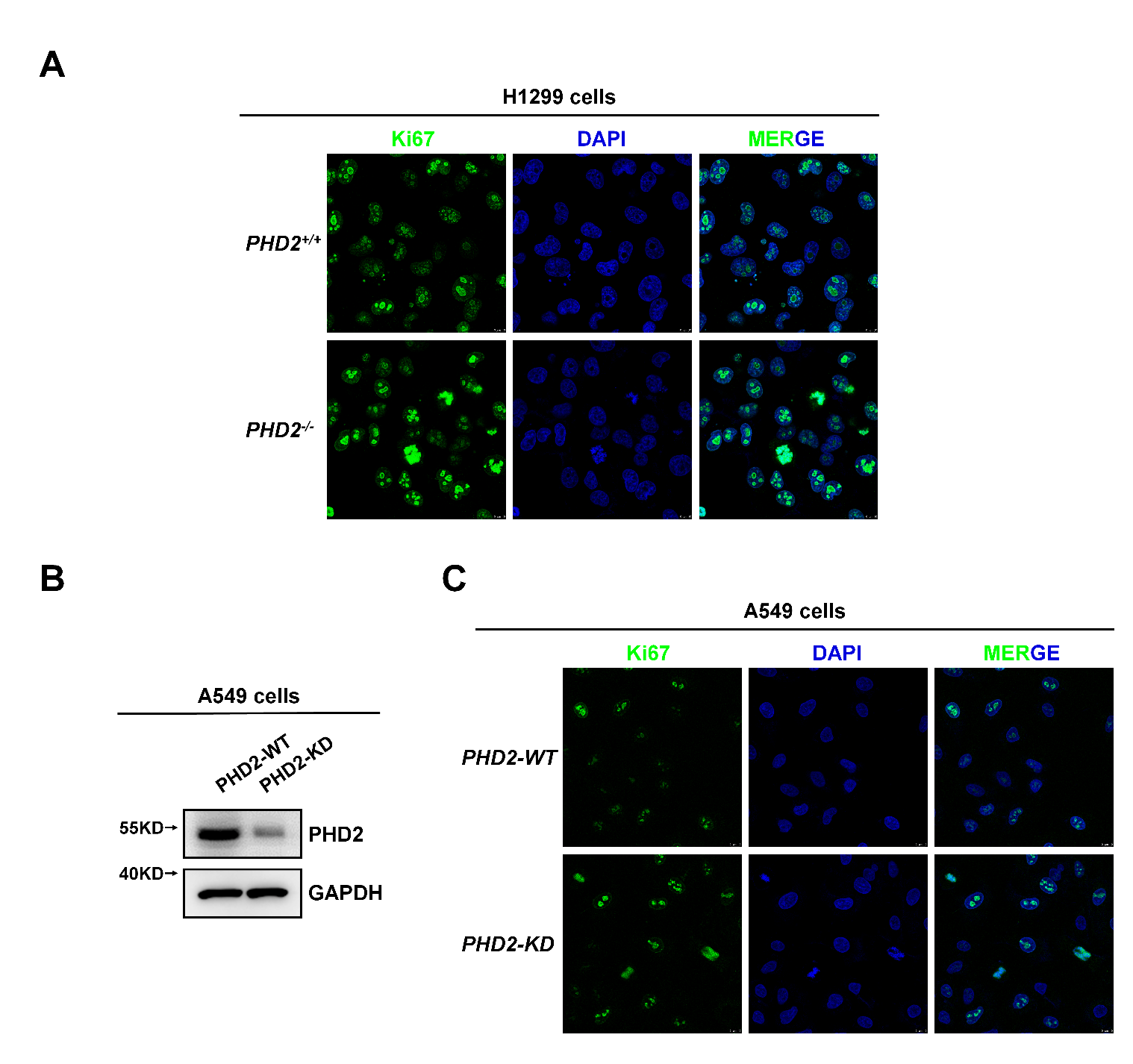


**Fig. S2.** **The expression of the proliferative marker Ki67 in PHD2-knockout H1299 cells or PHD2-knockdown A549 cells.**

(A) Immunofluorescence staining with anti-Ki67 antibody in wild-type (*PHD2^+/+^*) and PHD2-knockout (*PHD2^-/-^*) H1299 cells. Scale bar = 10 μm.

(B) Western blot analysis of endogenous PHD2 protein in wild-type (*PHD2-WT*) and PHD2-knockdown (*PHD-KD*) A549 cells.

(C) Immunofluorescence staining with anti-Ki67 antibody in wild-type (*PHD2-WT*) and PHD2-knockdown (*PHD-KD*) A549 cells. Scale bar = 10 μm.
